# Dazzle camouflage and the confusion effect: the influence of varying speed on target tracking

**DOI:** 10.1016/j.anbehav.2016.11.022

**Published:** 2017-01

**Authors:** Benedict G. Hogan, Innes C. Cuthill, Nicholas E. Scott-Samuel

**Affiliations:** a Biological Sciences, University of Bristol, Bristol, U.K.; b Experimental Psychology, University of Bristol, Bristol, U.K.

**Keywords:** animal groups, confusion effect, dazzle camouflage, defensive coloration, target tracking

## Abstract

The formation of groups is a common strategy to avoid predation in animals, and recent research has indicated that there may be interactions between some forms of defensive coloration, notably high-contrast ‘dazzle camouflage’, and one of the proposed benefits of grouping: the confusion effect. However, research into the benefits of dazzle camouflage has largely used targets moving with constant speed. This simplification may not generalize well to real animal systems, where a number of factors influence both within- and between-individual variation in speed. Departure from the speed of your neighbours in a group may be predicted to undermine the confusion effect. This is because individual speed may become a parameter through which the observer can individuate otherwise similar targets: an ‘oddity effect’. However, dazzle camouflage patterns are thought to interfere with predator perception of speed and trajectory. The current experiment investigated the possibility that such patterns could ameliorate the oddity effect caused by within-group differences in prey speed. We found that variation in speed increased the ease with which participants could track targets in all conditions. However, we found no evidence that motion dazzle camouflage patterns reduced oddity effects based on this variation in speed, a result that may be informative about the mechanisms behind this form of defensive coloration. In addition, results from those conditions most similar to those of published studies replicated previous results, indicating that targets with stripes parallel to the direction of motion are harder to track, and that this pattern interacts with the confusion effect to a greater degree than background matching or orthogonal-to-motion striped patterns.

## Highlights

• Variation in speed in groups induces the oddity effect, reducing predator confusion.
• Dazzle camouflage does not ameliorate this oddity effect.
• Parallel striped targets in groups are harder to track than other targets.

The formation of groups is a common strategy to avoid predation in animals, and recent research has indicated that there may be interactions between the benefits of grouping and those of defensive coloration. Motion dazzle camouflage consists of geometric high-contrast coloration and is hypothesized to interfere with an observer's accurate perception of speed and trajectory (Hall et al., 2016, Hogan et al., 2016a, Hogan et al., 2016b, Hughes et al., 2014, Scott-Samuel et al., 2011, Stevens et al., 2008, Thayer, 1909). In a recent study, Hogan, Cuthill, et al. (2016) found that targets with stripes parallel to a target's direction of movement impeded the tracking of one target among many, and that this effect interacted positively with increases in group size. This indicates that some animal patterns may carry benefits for animals moving in groups. While research is increasing our understanding of how animal coloration, object tracking and movement interact, all dazzle camouflage research to date has involved targets moving at constant speed. In animal groups, it is implausible that all members would move at a perfectly constant and equal speed. This could be for a number of stochastic reasons, for instance wind, terrain or water currents, or due to individual differences in age, size, health, etc.

One benefit of group membership in animals is the confusion effect: this describes a decrease in predator attack success with increased prey group size (Krause and Ruxton, 2002, Miller, 1922). It is thought that this occurs because of an increased cognitive challenge with increasing numbers of distractors in selecting and tracking a target (Ioannou et al., 2008, Krakauer, 1995, Ruxton et al., 2007). There is good evidence of this effect from a number of behavioural and computational experiments (see Jeschke & Tollrian, 2007 for a review). Individual variation in speed might be predicted to increase the ease with which predators can track and attack prey items in groups; this derives from the natural corollary of the confusion effect, the oddity effect, which suggests that targets that mismatch other group members in some way will be easier to track than ones that do not (Landeau & Terborgh, 1986). For the case of variation in speed, an individual's speed could help to identify it, in effect making the individual ‘odd’ and thereby undermining the confusion effect. Research into collective movement has found that animals in groups modify their behaviour, including speed, towards the group's average, especially when under high predation risk (Bode et al., 2010, Herbert-Read et al., 2013, Szulkin et al., 2006). It has been argued that this evidence suggests that animals in groups act to minimize individual differences that could undermine the confusion effect. However, there are few empirical data on the influence of oddity in dynamic properties, such as speed, on predation.

Despite the recent increase in research on the possible benefits of motion dazzle camouflage (Halperin et al., 2016, Hogan et al., 2016b, Hughes et al., 2015, Murali and Kodandaramaiah, 2016, Stevens et al., 2011, Stevens et al., 2008, von Helversen et al., 2013), relatively little is known about the mechanisms through which high-contrast patterns may benefit moving animals. However, it has been suggested that motion dazzle camouflage may act through the manipulation of perceived speed (Hall et al., 2016, Murali and Kodandaramaiah, 2016, Scott-Samuel et al., 2011, von Helversen et al., 2013). If motion dazzle camouflage introduces inaccuracies in the observer's perception of object speed, any harm accrued through oddity in individual speed may be minimized. Hogan, Cuthill, et al.'s (2016) recent findings do not support the necessity for variation in speed for dazzle camouflage to be beneficial to animals in groups. However, in their experiment, object speed was constant, so it might have been the case that the accurate perception of speed was relatively unimportant for accurate target tracking. This also precluded target speed as a parameter to distinguish between target and distractors through the oddity effect, a strategy that may similarly rely on the accurate perception of speed. Therefore, any potential benefits to motion dazzle camouflage with respect to speed might have been overlooked.

Investigation into the influence of variation in speed on dazzle camouflage may also shed light on the mechanisms underlying it. Two leading, although not mutually exclusive, hypotheses for the mechanisms of dazzle camouflage are the aperture problem and spatiotemporal aliasing (How and Zanker, 2014, Troscianko et al., 2009). The former arises because of the limited receptive field of individual visual motion receptors, and suggests that such receptors are unable to resolve ambiguity about the true direction of contours moving over their receptive field (Adelson and Movshon, 1982, Wuerger et al., 1996). Spatiotemporal aliasing arguments suggest that if the speed and spatial frequency of the contours on a moving object are well matched to the temporal and spatial sensitivity of the visual motion receptors, reversal of motion may occur when one contour is mistakenly identified as another (e.g. Pakarian & Yasamy, 2003). Either or both mechanisms could act to interrupt the accurate perception of speed. However, for patterns with contours orthogonal to the direction of movement, the spatiotemporal aliasing hypothesis would predict aberrant motion signals to occur mainly in opposition to true motion (How & Zanker, 2014). Since at some point local motion signals may be integrated (Saleem et al., 2012, Santer, 2013), it could be the case that such opposing motion signals would interrupt the perception of speed to a greater degree than motion signals from other patterns. Therefore, the spatiotemporal aliasing arguments could predict that the effects of the addition of variation in speed should differ between orthogonally striped and parallel striped patterns.

The current experiment aimed to address the importance of object speed in tracking by introducing conditions in which the speed of the target and distractors differed between individuals and varied over time. The inclusion of variation in speed allowed us to examine how this parameter affected the ability of the observer to track an individual in the group. Comparison of the influence of speed variation on tracking for the high-contrast targets relative to low-contrast background-matching targets indicates whether dazzle camouflage could ameliorate oddity in individual speed. Comparison of the effects of speed variation between the orthogonally and parallel striped conditions may be informative about the underlying mechanisms of dazzle camouflage. We used humans as a model species, tracking artificial targets on a screen. This approach has allowed great strides in our understanding of how perception and cognition affect visual search and predation, because of the precise control over not only stimulus properties but also ‘predator’ location and motivation (e.g. Ruxton et al., 2007, Stevens et al., 2011, Stevens et al., 2008). While other factors will undoubtedly affect predation on groups in the wild, some of these being species specific, in order to control prey motion and measure its effect it is almost essential to use artificial targets under tightly controlled viewing conditions.

## Methods

A computer-driven task was created in MatLab (Mathworks, Natick, MA, U.S.A.), which followed a similar methodology to that of Hogan, Cuthill, et al. (2016) and used identical equipment. Each trial, subjects were presented with sets of 1, 10, 30 or 50 moving squares which were constrained within a central area on the screen (268 × 268 pixels). Each square was 32 × 32 pixels in size, and the direction of movement of all squares from one frame to the next can be described as a correlated random walk (see Hogan, Cuthill, et al., 2016). The participant's task was to track the movements of a predetermined target square with a mouse-controlled on-screen cursor until the end of a 5000 ms moving period. The Cartesian locations of the centre of the target square and centre of the cursor were recorded every 10 ms. The mean distance of the cursor from the target in pixels for the final 4000 ms of each trial was calculated and recorded. Participants completed six practice trials which were excluded from the analysis, followed by 336 trials in six randomly ordered blocks, one for each combination of target coloration and speed condition. The order of blocks and of trials within each block were randomized independently for each subject. There were 15 participants (nine female), who were recruited opportunistically, and each was reimbursed £7 for participation. Each gave their informed written consent in accordance with the Declaration of Helsinki, and the experiment was approved by the Ethical Committee of the Faculty of Science, University of Bristol.

In some trials the speed of the squares was varied, with a maximum speed of 300 pixels/s and a minimum of 100 pixels/s. Each square was given an initial speed determined by the addition of a random value chosen from a normal distribution with a standard deviation of 40 and a mean of 0 to the average speed of 200 pixels/s. Each frame, each square's speed was independently determined by the addition of a random value chosen from an identical distribution to the square's speed in the previous frame. This meant that squares' speeds could differ from those of other group members, and that all squares' speeds changed over time. That is, our squares were not moving as a coordinated ‘herd’ or ‘shoal’; they were a milling swarm. This also meant that the average speed of each square over a trial was similar to that of squares that had constant speed, but the variance was much larger. In trials where speed was constant, each square's speed was fixed at 200 pixels/s.

**Figure 1:**
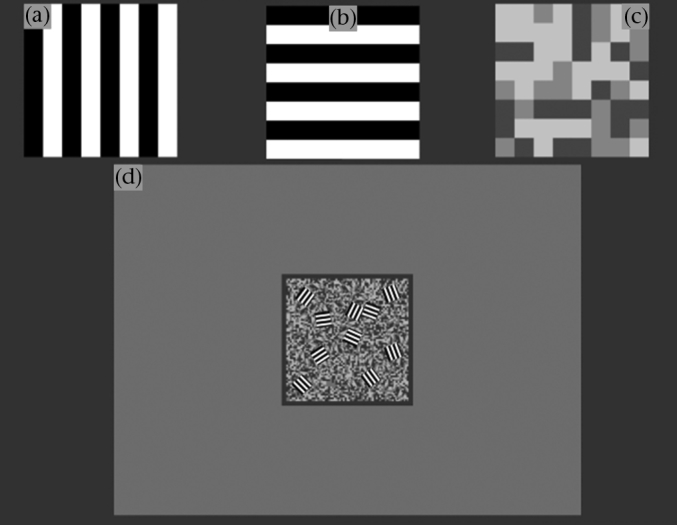
Illustration of the stimuli used. (a) Square pattern with stripes orthogonal to horizontal movement, (b) square pattern with lines parallel to horizontal movement, (c) trinary noise square pattern, (d) example of screen with striped targets on the trinary background.

Each trial, the background upon which the objects were drawn was a trinary noise pattern (see Hogan, Cuthill, et al., 2016). There were three coloration treatments applied to the moving squares: one was a trinary pattern created in the same way as the background coloration (see Fig. 1c). The other two were made up of a 100% contrast square-wave grating with wavelength 8 pixels, oriented either orthogonal or parallel in relation to the square's motion (see Fig. 1a and b).

All statistical analysis was performed in R (R Foundation for Statistical Computing, www.R-project.org). Participant mean response errors were distributed approximately log-normally, so were transformed with a natural logarithm for all analyses. The analysis followed the methods utilized in Hogan, Cuthill, et al. (2016), and used general linear mixed models (GLMM; function lmer in the lme4 package; Bates et al., 2015) with subject as a random factor. The most complex model fitted number of distractors as a quadratic polynomial, along with the two factors, target coloration type and speed variation condition. The first model included the three-way interaction of these factors, and subsequent models addressed whether main or interaction effects could instead be modelled as linear terms. The change in deviance between models with and without the predictor variables of interest was tested against a chi-square distribution with degrees of freedom equal to the difference in degrees of freedom between the models (Crawley, 2007).

## Results

The most complex model fitted number as a quadratic polynomial along with all interactions; this model fitted the data significantly better than a similar one that had a linear fit of number (X^2^_6_ = 59.9, *P* < 0.001) so all subsequent models fitted number as a quadratic polynomial. The three-way interaction between number, target coloration condition and speed variation was not significant (X^2^_4_ = 3.02, *P* = 0.554). There were significant two-way interactions between speed variation and number (X^2^_2_ = 6.90, *P* = 0.03) and between coloration condition and number (X^2^_4_ = 22.11, *P* < 0.001), but not between speed variation and coloration condition (X^2^_2_ = 2.48, *P* = 0.290). We therefore have no evidence that the effect of speed variation was modified by coloration.

**Figure 2:**
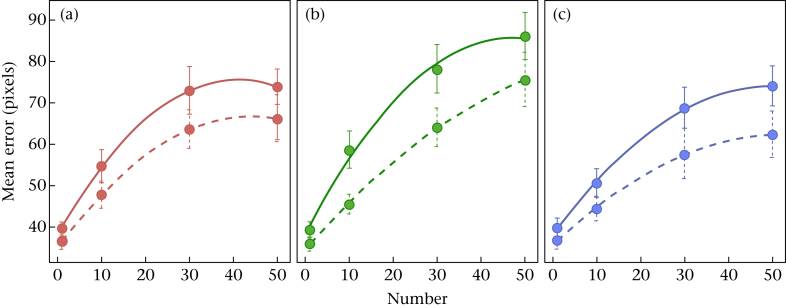
Individual plots of participant tracking for each background coloration against number of squares (target + distractors). (a) Orthogonal stripes, (b) parallel stripes, (c) trinary pattern. Solid lines indicate results when speed was constant; dashed lines indicate results when speed varied. Error bars indicate 95% confidence intervals for within-subject error.

The absence of a significant three-way interaction could result from lack of power, so it is useful to obtain an estimate of the effect size. The most sensitive test of our hypothesis that dazzle coloration ameliorates any oddity effect on target tracking is to examine the extent to which target colour pattern alters the effect of variable target speed in the treatment combination where the latter effect is greatest. This amounts to considering the difference between solid and dashed lines in Fig. 2, for all three pattern treatments, at 30 items, and estimating the differences between these. Parameter estimates with 95% confidence intervals (CIs) were calculated from the speed variation by coloration condition interaction in a GLMM on the logged error for the 30 items condition only. This post hoc analysis has an elevated type I error rate but the motivation is avoiding a type II error and, in any case, it is parameter estimation rather than null hypothesis testing that is of interest (Nakagawa & Cuthill, 2007). Comparing the orthogonally striped pattern with the trinary pattern, the estimate was 1.04 (95% CI: 0.96–1.13). Comparing the parallel striped pattern with the trinary pattern, the estimate was 0.98 (95% CI: 0.91–1.06). That is, the oddity effect due to speed variation (difference between solid and dashed lines in Fig. 2) observed in the trinary pattern treatment was, on average, changed by an orthogonally striped pattern by somewhere between a 4% increase and 13% reduction (mean 4% reduction) and for a parallel striped pattern by somewhere between a 9% increase and 6% reduction (mean 2% enhancement).

The significant interaction between speed variation and number indicates that the effects of speed variation changed with group size (see Fig. 2). Comparisons of the fitted parameters of the interaction indicate that the quadratic term of the slope of error against number was significantly higher when speed was constant than when it varied (*t*_14_ = 2.22, *P* = 0.043). This means that the slope of error against number was significantly more curved when speed was constant; that is, the effect of speed variation was relatively greater at intermediate than high group sizes (see Fig. 2). The slope of error against number when speed was constant was also nonsignificantly steeper than when speed varied (*t*_14_ = −1.33, *P* = 0.205).

The significant interaction between coloration condition and number indicates that the confusion effect was influenced by coloration condition. Comparison of the fitted linear terms of the interaction indicate that the relationship between error and number was significantly steeper in the parallel striped condition than in the orthogonally striped condition (*t*_14_ = 3.27, *P* = 0.006), and the trinary condition (*t*_14_ = −3.99, *P* = 0.001). The relationship between error and number did not differ significantly between the trinary and orthogonally striped conditions (*t*_14_ = −0.72, *P* = 0.483). This indicates that parallel striped conditions interacted with the confusion effect to a greater degree than the other coloration conditions, in line with the results of Hogan, Cuthill, et al. (2016). Comparisons of the fitted quadratic terms of the relationship between error and number for each coloration condition were not significant (orthogonal versus parallel: *t*_14_ = 1.2, *P* = 0.250; orthogonal versus trinary: *t*_14_ = 1.94, *P* = 0.073; parallel versus trinary: *t*_14_ = 0.74, *P* = 0.472).

## Discussion

The trend of increasing tracking error with group size in all conditions is in line with the confusion effect: there were greater errors in target tracking in larger groups, although the benefits (to the prey) dropped off with the largest group sizes. There was reduced tracking error in conditions where speed varied for all colour patterns, indicating that variation in interindividual speed may be costly to animals moving in groups because of the oddity effect. In addition, there was a significant interaction between coloration and number: the increase in error with group size was greater in parallel striped conditions than in the other conditions. In line with the results of Hogan, Cuthill, et al. (2016), this indicates that parallel striped colorations interacted with the confusion effect to a greater degree than orthogonally striped or trinary ones.

The addition of variation in speed apparently increased the ease with which participants could track a target individual in a group. This is not what one would expect from an advantage to ‘protean’ (unpredictable; Humphries & Driver, 1970) behaviour, and the fact that variability in angular direction increases the confusion effect (Scott-Samuel, Holmes, Baddeley, & Cuthill, 2015). This could be because in the current study individual speed became a cue to identity. Even though a target's speed varied, it did so in a correlated manner (and was uncorrelated with the speeds of neighbours), so it became a characteristic of a target item that was not shared by its neighbours. This helped, we propose, to disambiguate identity, consistent with the oddity effect (Landeau & Terborgh, 1986). This finding supports suggestions from research into collective motion that animals in groups should modify their speed to match that of the group to avoid predation (Bode et al., 2010, Herbert-Read et al., 2013, Szulkin et al., 2006). Note that in the current experimental design, variation in speed was not a simple case where distractors were identical and all different from the target. In this design, all targets varied in speed through identical mechanisms, but targets' instantaneous speed could differ from that of the distractors, and was autocorrelated such that a target's speed was likely to be similar to its speed in the previous time step. Indeed, our experiment modelled a swarm rather than a shoal; the targets moved in random and uncorrelated directions, which should make discriminations based on speed even more difficult. The large effect of speed variation found therefore indicates that participants were surprisingly sensitive to this nuanced and dynamic difference between the target and distractors. This suggests that for animals moving in groups, even if that movement is uncoordinated, departure from the speed of your neighbours may significantly increase predation risk.

Our results were not consistent with the hypothesis that motion dazzle camouflage patterns could provide an advantage through the manipulation of perceived speed. If this were the case, we would predict that the influence of speed variation would be reduced for these patterns relative to the trinary pattern, but we did not find evidence of this. Where the oddity effect was largest (30 items), the effect of parallel stripes, if anything, was to produce a modest increase in the effect of speed variation. For orthogonal stripes, the effect, if anything, was to produce a modest amelioration. Our experiment is therefore sufficiently powered to conclude that if there is an effect of pattern, it is relatively small for orthogonal and parallel stripes, which otherwise show the greatest effect (Fig. 2), the odds favouring no or a modest increase in the oddity effect. This may indicate that the mechanism through which parallel striped conditions interact with the confusion effect does not include manipulation of perceived speed. Further, our overall results are not consistent with the prediction from the hypothesized spatiotemporal aliasing mechanism for dazzle camouflage that the influence of speed variation should differ significantly between parallel and orthogonally striped patterns. Additionally, the benefits to prey of parallel striped targets that we found are not easily reconciled with either spatiotemporal aliasing or aperture problem mechanisms for dazzle camouflage. As in the experiment by Hogan, Cuthill, et al. (2016) and in contrast to several previous experiments on dazzle camouflage, targets and distractors in this experiment moved in an unpredictable fashion with many turns. This could mean that correct assessment of the heading and rotation of targets is of heightened importance in these studies. It could be that these represent the parameters that are influenced by parallel striped conditions (Hogan, Cuthill, et al., 2016). Several of the empirical experiments on dazzle camouflage have focused on the hypothesis that bias in perceived speed could represent the primary mechanism by which this type of coloration reduces predation risk (Hall et al., 2016, Hogan et al., 2016a, Murali and Kodandaramaiah, 2016, von Helversen et al., 2013). Our results indicate that misperception in speed may not always drive the benefits of dazzle camouflage.

The effect of speed variation was relatively greater in intermediate than high group sizes. This may indicate that the influence of oddity is disproportionately small when confusion is high. The effect of group size on oddity has received some attention in previous experimentation on the confusion effect. In a neural network, Krakauer (1995) found that oddity reduced confusion to a greater degree in larger than in smaller groups. However, in a computer-based behavioural experiment with human participants Ruxton et al. (2007) found no interaction between group size and the effects of target oddity. Further behavioural research on the oddity effect may help us to understand how and whether the influence of oddity changes with group size. This may allow us to make predictions about the expected composition and behaviour of animal species benefiting from the confusion effect.

The overall pattern of results when speed was constant corresponds well with those of Hogan, Cuthill, et al. (2016): parallel striped targets were significantly harder to track, and interacted with the confusion effect to a greater degree than the other patterns. Further, targets whose speed varied were easier to catch than those with steady speed, a finding that indicates that animals in groups do not benefit from randomness in speed (in contrast to randomness in direction) and may better impede tracking by matching the speed of their neighbours. Our findings were not consistent with the suggestion that high-contrast dazzle patterns could act to minimize the costs to prey of departure from the speed of their neighbours, a result that could be informative about the mechanisms of dazzle camouflage for animals moving in groups.
